# Supplementary material for: Malaria parasites use a soluble RhopH complex for erythrocyte invasion and an integral form for nutrient uptake
Source: eLife. 2021 Jan 4;10:e65282. doi: 10.7554/eLife.65282 (PMC7840181; doi:10.7554/eLife.65282)
Supplement: Transparent reporting form [file elife-65282-transrepform.pdf]

eLIFE
1st Floor
24 Hills Road
Cambridge CB2 1JP, UK
P 01223 855340
W elifesciences.org
T @elife

eLife's transparent reporting form

We encourage authors to provide detailed information *within their submission* to facilitate the interpretation and replication of experiments. Authors can upload supporting documentation to indicate the use of appropriate reporting guidelines for health-related research (see EQUATOR Network), life science research (see the BioSharing Information Resource), or the ARRIVE guidelines for reporting work involving animal research. Where applicable, authors should refer to any relevant reporting standards documents in this form.

If you have any questions, please consult our Journal Policies and/or contact us: editorial@elifesciences.org.

Sample-size estimation

- You should state whether an appropriate sample size was computed when the study was being designed
- You should state the statistical method of sample size computation and any required assumptions
- If no explicit power analysis was used, you should describe how you decided what sample (replicate) size (number) to use

Please outline where this information can be found within the submission (e.g., sections or figure legends), or explain why this information doesn't apply to your submission:

- The sample size for cryo-EM analysis was chosen to obtain the best possible resolution. We maximized the number of particles collected based on sample and microscope availability to permit *de novo* model building and the highest achievable resolution. The sample sizes and procedures are detailed in the Materials and Methods and Figure 2—figure supplement 1.
- No sample size was computed during study design.
- No statistical method of sample size computation was performed.
- Membrane fractionation studies were performed in three independent biological replicates after an optimal procedure was defined. Sample sizes for each experiment are indicated in the Figure 4 legend. All data were included without exclusion.

Replicates

- You should report how often each experiment was performed
- You should include a definition of biological versus technical replication
- The data obtained should be provided and sufficient information should be provided to indicate the number of independent biological and/or technical replicates
- If you encountered any outliers, you should describe how these were handled
- Criteria for exclusion/inclusion of data should be clearly stated
- High-throughput sequence data should be uploaded before submission, with a private link for reviewers provided (these are available from both GEO and ArrayExpress)

Please outline where this information can be found within the submission (e.g., sections or figure legends), or explain why this information doesn't apply to your submission:

- Multiple negative stain imaging experiments were performed to optimize harvest and imaging of the RhopH complex. With these optimized conditions, a single large protein purification was used for cryo-EM data acquisition based on sample and microscope availability constraints.
- Cryo-EM analysis identified 68,216 well-ordered particles through generally accepted procedures for 2D and 3D classification, as detailed in the Materials and Methods and in Figure 2—figure supplement 1.
- Each membrane fractionation experiment was performed with three independent biological replicates, defined as independent culture harvests and biochemical preparations. No data were excluded ;no outlier analysis was required or performed.

Statistical reporting

- Statistical analysis methods should be described and justified
- Raw data should be presented in figures whenever informative to do so (typically when N per group is less than 10)
- For each experiment, you should identify the statistical tests used, exact values of N, definitions of center, methods of multiple test correction, and dispersion and precision measures (e.g., mean, median, SD, SEM, confidence intervals; and, for the major substantive results, a measure of effect size (e.g., Pearson's r, Cohen's d)
- Report exact p-values wherever possible alongside the summary statistics and 95% confidence intervals. These should be reported for all key questions and not only when the p-value is less than 0.05.

Please outline where this information can be found within the submission (e.g., sections or figure legends), or explain why this information doesn't apply to your submission:

- Cryo-EM map resolution was determined using the gold-standard Fourier shell correlation method using two independently refined half-maps after random allocation of particles to two groups. Details are provided in the Materials and Methods.
- A representative CTF-corrected cryo-EM micrograph is shown Figure 2—figure supplement 1.
- Mean and standard error values from three biological replicates are reported for each membrane fractionation experiment. Statistical analysis was performed using unpaired Student's *t* test or one-way ANOVA with posthoc Tukey's multiple comparisons test as appropriate; *P* values are reported in the Figure 4 legend.
- Representative western blots are shown for each experiment in Figure 4.

(For large datasets, or papers with a very large number of statistical tests, you may upload a single table file with tests, Ns, etc., with reference to sections in the manuscript.)

Group allocation

- Indicate how samples were allocated into experimental groups (in the case of clinical studies, please specify allocation to treatment method); if randomization was used, please also state if restricted randomization was applied
- Indicate if masking was used during group allocation, data collection and/or data analysis

Please outline where this information can be found within the submission (e.g., sections or figure legends), or explain why this information doesn't apply to your submission:

No experimental grouping was performed.

Additional data files ("source data")

- We encourage you to upload relevant additional data files, such as numerical data that are represented as a graph in a figure, or as a summary table
- Where provided, these should be in the most useful format, and they can be uploaded as "Source data" files linked to a main figure or table

- Include model definition files including the full list of parameters used
- Include code used for data analysis (e.g., R, MatLab)
- Avoid stating that data files are "available upon request"

Please indicate the figures or tables for which source data files have been provided:

N/A
